# Biodegradation of Chlorpyrifos and Its Hydrolysis Product 3,5,6-Trichloro-2-Pyridinol by a New Fungal Strain *Cladosporium cladosporioides* Hu-01

**DOI:** 10.1371/journal.pone.0047205

**Published:** 2012-10-08

**Authors:** Shaohua Chen, Chenglan Liu, Chuyan Peng, Hongmei Liu, Meiying Hu, Guohua Zhong

**Affiliations:** Laboratory of Insect Toxicology, South China Agricultural University, Guangzhou, People’s Republic of China; Naval Research Laboratory, United States of America

## Abstract

Intensive use of chlorpyrifos has resulted in its ubiquitous presence as a contaminant in surface streams and soils. It is thus critically essential to develop bioremediation methods to degrade and eliminate this pollutant from environments. We present here that a new fungal strain Hu-01 with high chlorpyrifos-degradation activity was isolated and identified as *Cladosporium cladosporioides* based on the morphology and 5.8S rDNA gene analysis. Strain Hu-01 utilized 50 mg·L^−1^ of chlorpyrifos as the sole carbon of source, and tolerated high concentration of chlorpyrifos up to 500 mg·L^−1^. The optimum degradation conditions were determined to be 26.8°C and pH 6.5 based on the response surface methodology (RSM). Under these conditions, strain Hu-01 completely metabolized the supplemented chlorpyrifos (50 mg·L^−1^) within 5 d. During the biodegradation process, transient accumulation of 3,5,6-trichloro-2-pyridinol (TCP) was observed. However, this intermediate product did not accumulate in the medium and disappeared quickly. No persistent accumulative metabolite was detected by gas chromatopraphy-mass spectrometry (GC-MS) analysis at the end of experiment. Furthermore, degradation kinetics of chlorpyrifos and TCP followed the first-order model. Compared to the non-inoculated controls, the half-lives (*t*_1/2_) of chlorpyrifos and TCP significantly reduced by 688.0 and 986.9 h with the inoculum, respectively. The isolate harbors the metabolic pathway for the complete detoxification of chlorpyrifos and its hydrolysis product TCP, thus suggesting the fungus may be a promising candidate for bioremediation of chlorpyrifos-contaminated water, soil or crop.

## Introduction

Synthetic organophosphates (OPs) are the most frequently and widely used insecticides, accounting for an estimated 34% of world-wide insecticide sales [1], [2]. Most organophosphorus insecticides share a similar structure, containing three phosphoester linkages and are hence often termed phosphotriesters [3]. This class of pesticides have acute neurotoxicity attributing to their ability to suppress acetylcholinesterase (AchE) [4], and various clinical effects can occur due to their poisoning in human beings [5]–[9].

**Figure 1:**
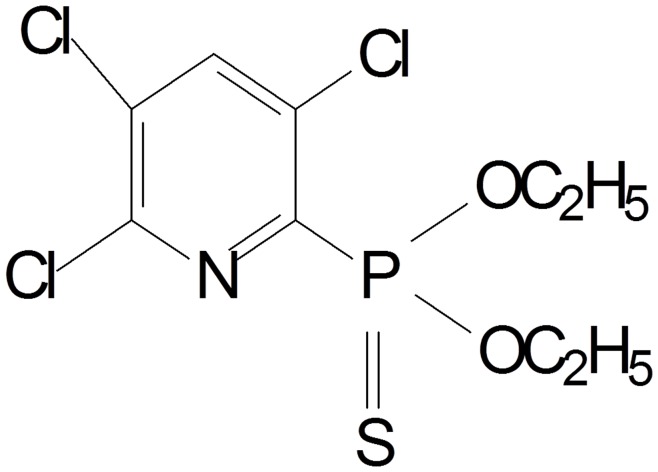
Molecular structure of chlorpyrifos.

Chlorpyrifos, [*O*,*O*-diethyl *O*-(3,5,6-trichloro-2-pyridyl) phosphorothioate] is one of the most extensively used broad-spectrum OPs, and its phosphorus is linked to a sulfur with a double bond (P = S) (Fig. 1). It is used throughout the world to control a variety of chewing and sucking insect pests and mites on a range of economically important crops, including citrus fruit, bananas, vegetables, potatoes, coffee, cocoa, tea, cotton, wheat, rice, and so on [10]. It is also registered for use on lawns, ornamental plants, animals, domestic dwellings as well as commercial establishments [11].

A consequence of the persistent usage and broad-spectrum applicability of chlorpyrifos is widespread contamination in natural environment, leading to serious damage to non-target organisms [12]–[14]. Previous reports revealed that a wide range of water and terrestrial ecosystems have been contaminated with chlorpyrifos [15]–[17]. Moreover, much evidence suggests that chlorpyrifos may affect the endocrine system, respiratory system, cardiovascular system, nervous system, immune system, as well as the reproductive system due to its high mammalian toxicity [18]–[27]. Additionally, chlorpyrifos can be converted to 3,5,6-trichloro-2-pyridinol (TCP) in natural environment, a persistent metabolite that is refractory to microbial degradation [28], [29]. It has been suggested that the accumulated TCP in liquid medium or soil, which has antimicrobial property, prevents the proliferation of microorganisms involving in degrading chlorpyrifos [30]. TCP is classified as persistent and mobile by the US Environmental Protection Agency (EPA) with a half life ranging from 65 to 360 d in soil, depending on the soil type, climate, and other conditions [30]. As the major degradation product of chlorpyrifos, TCP has greater water solubility than its parent molecule and causes widespread contamination of soils and aquatic environments [4], [29], [30]. It is therefore essential to eliminate these pollutants from the environments.

Microbial detoxification of chlorpyrifos has become the focus of many studies because other methods of removing chlorpyrifos residues are impractical or costly or are themselves environmentally hazardous [5]. To date, several chlorpyrifos-degrading bacterial strains including *Enterobacter* strain B-14 [31], *Stenotrophomonas* sp. strain YC-1 [32], *Sphingomonas* sp. strain Dsp-2 [33], *Paracoccus* sp. strain TRP [29], *Bacillus pumilus* strain C2A1 [4], and *Bacillus laterosporus* strain DSP [34] have been isolated from diverse sources; however, only *Paracoccus* sp. strain TRP and *Bacillus pumilus* strain C2A1 were able to degrade both chlorpyrifos and TCP. One recently isolated cyanobacterium, *Synechocystis* sp. strain PUPCCC 64, was also capable of degrading chlorpyrifos [35]. However, there is limited information concerning the ability of fungus to degrade chlorpyrifos, e.g. only *Verticillium* sp. strain DSP [36], [37] and *Acremonium* sp. strain GFRC-1 [38]. Fungi are critical to the biogeochemical cycle and are responsible for the bulk of the degradation of environmental xenobiotics in the biosphere [39]. Moreover, the ability of fungi to form extended mycelial networks, the low specificity of their catabolic enzymes and their independence from utilizing organic chemicals as a growth substrate make fungi well suited for bioremediation processes [40]. However, the potential use of fungus in bioremediation of OPs has not received the attention it deserves. This is the first report to our knowledge involving in the biodegradation of both chlorpyrifos and its hydrolysis product TCP by fungus.

In the current study, a new fungus *Cladosporium cladosporioides* strain Hu-01 able to degrade not only chlorpyrifos but also TCP was isolated and characterized. Moreover, the optimal culture conditions were investigated and the degradation pathway was elucidated.

## Materials and Methods

### Chemicals and Media

Chlorpyrifos standard (97% purity) was obtained from Dow AgroSciences, USA. TCP standard (99% purity) was purchased from Sigma-Aldrich, USA. Chromatographic-grade methanol was purchased from Burdic & Jackson, USA. All other chemicals and reagents used were of pure analytical-grade and available commercially. Stock solutions (10 g·L^−1^) were prepared with methanol, sterilized by membrane filtration (0.45 µm), and stored in dark bottles at 20°C before use.

Czapek-Dox medium (CDM) containing (in gram per litre) sucrose, 30; NaNO_3_, 2; KCl, 0.5; MgSO_4_, 0.5; K_2_HPO_4_, 1; Fe_2_(SO_4_)_3_, 0.01; and peptone, 0.5; and mineral salt medium (MSM) containing (in gram per litre) (NH_4_)_2_SO_4_, 2; MgSO_4_·7H_2_O, 0.2; CaCl_2_·2H_2_O, 0.01; FeSO_4_·7H_2_O, 0.001, Na_2_HPO_4_·12H_2_O, 1.5; and KH_2_PO_4_, 1.5 were used for the isolation of fungal strains.

### Enrichment and Isolation of the Chlorpyrifos-degrading Fungi

Activated sludge samples were collected as the inoculum from an aerobic chlorpyrifos-manufacturing wastewater treatment system located in Jiangmen, China, which had produced chlorpyrifos for over 15 years. Enrichment and isolation of degrading strains were carried out in MSM by using an enrichment culture technique as described in detail previously [41]–[43]. Colonies with different morphologies grown on the plates were picked and purified using the streaking method. The isolates were tested for their capacities to degrade chlorpyrifos and TCP. One pure isolate showing the highest degradation was selected for further study and designated Hu-01.

### Identification and Characterization of Strain Hu-01

The isolate was grown on CDM agar plates for 5 d and its morphology was investigated by light microscope (BH-2 Olympus, Japan) and scanning electron microscope (XL-30 ESEM, Philips Optoelectronics Co., Ltd, Holland). Colony morphology was observed on CDM agar plates incubated at 27°C at 1, 2, 3, 4, and 5 d. Total genomic DNA was prepared according to the method of Sambrook and Russell [44]. The 5.8S rDNA gene was amplified by polymerase chain reaction (PCR) with the universal primers ITS 5 (5′-GGAAGTAAAAGTC GTAACAAGG-3′) and ITS 4 (5′-GCATATCAATAAGCGGAGGA-3′) [45]. Reaction conditions consisted of initial denaturation at 94°C for 3 min, followed by 35 cycles of denaturation at 94°C for 30 s, annealing at 55°C for 1 min, and extension at 72°C for 1 min, with the last cycle followed by a ten-minute extension at 72°C. After purification by agarose gel electrophoresis, PCR fragments were ligated to the linearized vector pMD20-T (TaKaRa Biotechnology Co. Ltd., China), and transformed into competent *Escherichia coli* DH5α cells. Sequencing of the cloned insert was performed by Shanghai Invitrogen Technology Co. Ltd., China. The resulting sequence was compared with the genes available in the GenBank nucleotide library by a BLAST search through the National Center for Biotechnology Information (NCBI) internet site. Multiple alignments of 5.8S rDNA were performed by ClustalX 1.8.1 with default settings, and phylogenesis was analyzed using MEGA 4.0 software. An unrooted tree was built using the neighbor-joining method [46].

### Inoculum Preparation

Inoculum was prepared by growing the isolate in 50 mL of CDM for 3 d at 27°C with shaking at 150 rpm. Mycelia were harvested by centrifugation at 4600 × g for 5 min, washed with 0.9% sterile saline and resuspended in 50 mL saline. Two percent of this suspension was used as inoculum for chlorpyrifos and TCP biodegradation studies [47].

### Optimization of the Culture Conditions for Chlorpyrifos Degradation by Strain Hu-01

The culture conditions favoring chlorpyrifos degradation by strain Hu-01 was determined by using response surface methodology (RSM) [48]. The significant factors that were selected as independent variables were pH, temperature, and culture time based on the results of preliminary one-factor-at-a-time experiments. A five-level (−1.68, −1, 0, 1, 1.68) central composite rotatable design (CCRD) consisting of 23 experimental runs with three replicates at the center point was generated by statistic analysis system (SAS) software package (Version 9.0, SAS Institute Inc., Cary, NC, USA). Regression analysis was performed on the data obtained from the design experiments. Coding of the variables was carried out according to the following equation (Eq.(1))
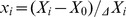
(1)where *x*_i_ is the dimensionless value of an independent variable, *X*_i_ is the real value of an independent variable, *X*_0_ is the real value of an independent variable at the center point, and _Δ_ *X*_i_ is the step change of real value of the variable *i* corresponding to a variation of a unit for the dimensionless value of the variable *i*. The symbols and levels of the three independent variables are presented in Table 1. The dependent variable was the degradation of 50 mg·L^−1^ chlorpyrifos by strain Hu-01 by hour 12. Randomised block design was conducted in order to minimise the effects of unexplained variability in the observed response because of extraneous factors [49]. The data were analyzed by using the response surface regression procedure of the SAS software to fit the following quadratic polynomial equation (Eq.(2))
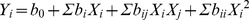
(2)where Yi is the predicted response, Xi and Xj are variables, b0 is the constant, bi is the linear coefficient, bij is the interaction coefficient, and bii is the quadratic coefficient, respectively.

**Table 1:** The symbols and levels of three independent variables used in central composite rotatable design (CCRD).

Independent variables	Symbol	Code levels of variables				
		−1.68	−1	0	1	1.68
pH	X_1_	3.97	5.00	6.50	8.00	9.02
Temperature (°C)	X_2_	23.30	25.00	27.50	30.00	31.70
Culture time (d)	X_3_	2.32	3.00	4.00	5.00	5.68

The coefficient of determination (*R* ^2^) and adjusted *R* ^2^ (Adj.*R* ^2^) were used for the verification of the significance of the quadratic polynomial model. The significant variables were screened based on the *F*-test and the *P*-value at the 95% significance level [50]. On the basis of the analysis of variance (ANOVA), parameters with a significance level (*P*) greater than 5% were removed to obtain the final reduced model. The final model can be displayed as three-dimensional (3D) response surface plots by varying two factor levels while keeping the other factor at a constant level [51]. The SAS software package was applied for the regression analysis and the graphical presentation.

### Identification of Metabolic Products during Chlorpyrifos Degradation

The degradation products of chlorpyrifos in mycelium-free filtrates of the fungal cultures grown in MSM containing 50 mg·L^−1^ of chlorpyrifos were determined by gas chromatopraphy-mass spectrometry (GC-MS) (Agilent 6890N/5975, USA). The mycelium-free filtrates were collected at 1, 2, 3, 4, 5, and 6 d, respectively. Non-inoculated media served as controls. The metabolic products confirmed on the basis of mass spectrum were matched with authentic standard compounds from the National Institute of Standards and Technology (NIST, USA) library database.

### Degradation Kinetics of Chlorpyrifos and TCP by Strain Hu-01

The abilities of strain Hu-01 to degrade chlorpyrifos and its hydrolysis product TCP were investigated in MSM under the optimal culture conditions. Chlorpyrifos and TCP were added to 250-ml Erlenmeyer flasks to give the final concentration of 50 mg·L^−1^, respectively. The flasks were incubated at 27°C on a platform shaker at 150 rpm for 7 d. The experiment was conducted in triplicate with non-inoculated samples as control. The samples were collected periodically and the chemical residues were measured by high performance liquid chromatography (HPLC) (Agilent 1100, USA).

### Chemical Analysis

A modified method from Gao et al. [52] was used to determine the residues of chlorpyrifos and TCP. In brief, samples (30 mL) were extracted using the mixture of acetone and dichloromethane in an equal volume ratio (1∶1, *v/v*). After concentration by using rotary evaporator (Heidolph, Germany), residues dissolved in methanol were determined by HPLC equipped with a Hypersil ODS2 C_18_ reversed phase column (4.6 nm × 250 mm, 5 µm) with array detection from 190–400 nm (total scan). A mixture of methanol and water (70∶30, *v/v*) was used as the mobile phase after acidification to pH 3 with concentrated phosphoric acid. The injection volume was 10 µL.

The degradation products of chlorpyrifos were identified according to the method of Xu et al. [29] with modification. In brief, the mycelium-free cultures were extracted with petroleum ether and the supernatant was dehydrated, dried and re-dissolved in methanol. After filtration with 0.45 µm membrance (Millipore, USA), the samples were detected by GC-MS system equipped with auto-sampler, an on-column, split/splitless capillary injection system, and with HP-5MS capillary column (30.0 m × 250 µm × 0.25 µm) with array detection from 30–500 nm (total scan). The operating conditions were as follows: the column was held at 80°C for 5 min, ramped at 8°C·min^−1^ to 200°C (first ramp), held at 200°C for 5 min, ramped at 15°C·min^−1^ to 260°C (second ramp), and then held at at 260°C for 5 min. The temperatures corresponding to transfer line and the ion trap were 280°C and 230°C, respectively, and the ionization energy was 70 eV. The injection volume was 1.0 µL with a split ratio of 1∶7 at 260°C. Helium was used as a carrier gas at a flow rate of 1.0 mL·min^−1^.

### Data Analysis

Results were assessed by ANOVA and statistical analyses were performed on three replicates of data obtained from each treatment. The significance (*P*<0.05) of differences was treated statistically by one-, two-, or three-way ANOVA and evaluated by Tukey’s test.

### Ethics Statement

No specific permits were required for the described field studies. No specific permissions were required for these locations. We confirm that the location is not privately-owned or protected in any way. We confirm that the field studies did not involve endangered or protected species.

## Results

### Isolation and Identification of the Fungal Strain Hu-01

After five rounds of transfer, a total of 8 pure isolates able to grow with chlorpyrifos as the sole carbon source were obtained from the enrichment culture (Table S1). One fungal strain, designated Hu-01, was selected for further study due to its highest degradation activity on both chlorpyrifos and TCP. This fungus degraded and tolerated chlorpyrifos and TCP with concentrations as high as 500 mg·L^−1^, and completely metabolized the added chlorpyrifos and TCP (both 50 mg·L^−1^) within 5 and 6 d, respectively.

**Figure 2:**
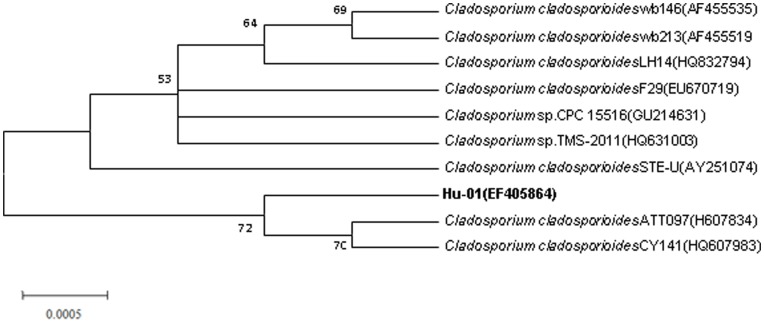
Phylogenetic tree based on the 5.8S rRNA sequence of strain Hu-01 and related strains. Numbers in parentheses represent the sequences accession number in GenBank. Numbers at the nodes indicate bootstrap values from the neighborhood-joining analysis of 1,000 resampled data sets. Bar represents sequence divergence.

Strain Hu-01 was an obligately aerobic fungus with strong spore production. Colonies were dark green, round, and with concentric rings and entire margin when grown on CDM agar plates. Spores were oval, umbilicate, and with dimensions of 4.0 to 6.0 µm in length and 2.0 to 3.0 µm in width (Figure S1). Mycelia were dark olivaceous which differed from most genus of fungi. A single fragment of 574 bp was obtained after the PCR amplification. The DNA G+C content is 50.9 mol %. Phylogenetic analysis of the 5.8S rDNA gene sequences revealed that strain Hu-01 grouped among *Cladosporium* species and was closely related to *C. cladosporioides* strain ATT097 (GenBank accession No. H607834) and *C. cladosporioides* strain CY141 (GenBank accession No. HQ607983) with high identities (>99%) (Fig. 2). Thus, strain Hu-01 was tentatively identified as *C. cladosporioides* based on the morphology and 5.8S rDNA gene analysis. The partial 5.8S rDNA gene sequences have been deposited at the GenBank under the accession No. EF405864. The *C. cladosporioides* strain Hu-01 was also deposited in China Center for Type Culture Collection under the collection No. CCTCC M 20711.

### Optimization of the Culture Conditions for Chlorpyrifos Degradation by Strain Hu-01

Response surface methodology (RSM) based on the central composite rotatable design (CCRD) was employed to investigate the main and interactive effects of significant variables including pH (*X*_1_), temperature (*X*_2_), and culture time (*X*_3_) on chlorpyrifos degradation by strain Hu-01. The design matrix of the variables together with the experimental responses is given in Table 2. Subsequently, the data from Table 2 were assessed by response surface regression procedure of SAS software package, and the results of the quadratic polynomial model fitting in the term of analysis of variance (ANOVA) were shown in Table 3. By applying the multiple regression analysis on the experimental data, the following quadratic polynomial equation (Eq.(3)) was derived to explain the chlorpyrifos degradation:
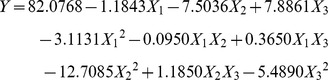
(3)where *Y* is the predicted chlorpyrifos degradation (%) by strain Hu-01; *X*_1_, *X*_2_, and *X*_3_ are the coded values for the pH, temperature, and culture time, respectively.

**Table 2:** Central composite rotatable design (CCRD) matrix and the response of dependent variable for chlorpyrifos degradation.

Run	Code levels of independent variables			Response
	*X*_1_	*X*_2_	*X*_3_	Chlorpyrifos degradation (%) *Y*
1	−1.00	−1.00	−1.00	63.62±0.63g
2	−1.00	−1.00	1.00	78.30±1.13b
3	−1.00	1.00	−1.00	50.97±1.11j
4	−1.00	1.00	1.00	68.17±0.93e
5	1.00	−1.00	−1.00	61.00±0.76h
6	1.00	−1.00	1.00	74.92±0.96c
7	1.00	1.00	−1.00	45.75±1.12l
8	1.00	1.00	1.00	66.63±0.82f
9	−1.68	0.00	0.00	70.18±1.10d
10	1.68	0.00	0.00	68.15±0.56e
11	0.00	−1.68	0.00	58.72±0.96i
12	0.00	1.68	0.00	25.33±0.68b
13	0.00	0.00	−1.68	50.25±0.93k
14	0.00	0.00	1.68	74.64±0.77c
15	0.00	0.00	0.00	84.18±0.46a
16	0.00	0.00	0.00	82.50±0.46a
17	0.00	0.00	0.00	80.11±0.47a
18	0.00	0.00	0.00	81.85±0.46a
19	0.00	0.00	0.00	81.44±0.45a
20	0.00	0.00	0.00	82.83±0.46a
21	0.00	0.00	0.00	80.87±0.44a
22	0.00	0.00	0.00	84.43±0.46a
23	0.00	0.00	0.00	81.90±0.45a

*X*_1_ refers to pH; *X*_2_ refers to temperature; *X*_3_ refers to culture time. The data presented are means of three replicates with standard deviation. Data followed by the same letters in the same column are not significantly different at *P* = 0.05 level according to the Tukey’s test.

The statistical significance of Eq.(3) was also evaluated by performing *F*-test (Table 3) and *t*-test (Table 4). The statistical analysis indicated that the model linear term coefficient of *X*_2_ and *X*_3_ and the quadric term coefficient of *X*_1_, *X*_2_ and *X*_3_ showed significant effects (*P*<0.05) on chlorpyrifos degradation. On the contrary, the linear term coefficient of *X*_1_, and the cross-product coefficients were not significant (*P*>0.05), so the quadratic polynomial equation (Eq.(4)) was modified to be:
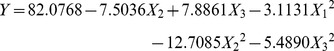
(4)


**Table 3:** Analysis of variance (ANOVA) for the fitted quadratic polynomial model for chlorpyrifos degradation.

Source	DF	SS	MS	*F* Value	*P*r > *F* ^*^
*X*_1_	1	19.1551	19.1551	0.9900	0.3379
*X*_2_	1	768.9248	768.9248	39.7411	0.0001
*X*_3_	1	849.3227	849.3227	43.8964	0.0001
*X*_1_ *X*_1_	1	153.9879	153.9879	7.9587	0.0144
*X*_1_ *X*_2_	1	0.0722	0.0722	0.0037	0.9522
*X*_1_ *X*_3_	1	1.0658	1.0658	0.0551	0.8181
*X*_2_ *X*_2_	1	2566.2370	2566.2370	132.6334	0.0001
*X*_2_ *X*_3_	1	11.2338	11.2338	0.5806	0.4597
*X*_3_ *X*_3_	1	478.7264	478.7264	24.7425	0.0002
Model	9	4821.4490	535.7165	27.6880	0.0001
Error	13	251.5286	19.3484		
Total	22	5072.9770			

*R* ^2^ = 0.9504; coefficient of variation (CV) = 5.8%; Adj.*R* ^2^ = 0.9285. DF refers to degrees of freedom; SS refers to sum of sequences; MS refers to mean square. ^*^ *P* Level less than 0.05 indicate the model terms are significant.

The adequacy of the model was examined by the determination coefficient (*R* ^2^ = 0.9650), which explained 96.50% of the response variability, suggesting that the predicted values of the model were well correlated with the experimental values (Table 4). The high value of the adjusted *R* ^2^ (0.9408) further supported the accuracy of the model. These results suggested that the response equation provided a suitable and reasonable model for the CCRD experiment. Besides, the low coefficient of variation (CV = 5.8%) demonstrated a good precision and reliability of the experiments. The developed model thus could be adequate for prediction within the range of variables employed.

**Figure 3:**
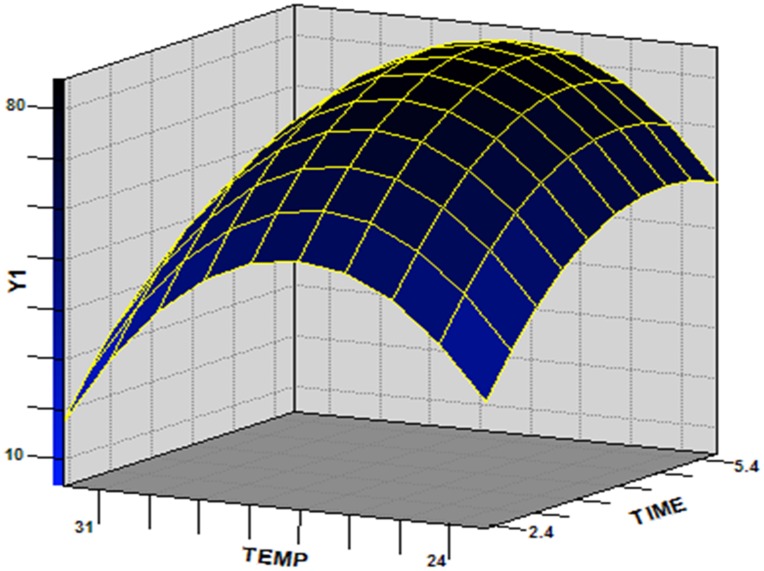
Response surface plot showing the effects of temperature and culture time on chlorpyrifos degradation by strain Hu-01 with pH = 6.5.

A three-dimensional (3D) response surface was plotted to directly display the effects of temperature and culture time on the chlorpyrifos degradation by strain Hu-01 while keeping the value of pH (the non-significant variable) at a zero level (Fig. 3). The model predicted a maximum chlorpyrifos degradation of 85.9% at the stationary point. At the stationary point, the optimum levels for the three variables of *X*_1_, *X*_2_ and *X*_3_ were observed to be −0.1460, −0.2627 and 0.6851 in terms of the coded units, that is, pH 6.5, temperature 26.8°C, and culture time 4.7 d, respectively. So the optimum culture conditions for chlorpyrifos degradation by strain Hu-01 were determined to be pH 6.5, 26.8°C, and 4.7 d.

**Table 4:** Effect estimates for the fitted quadratic polynomial model for chlorpyrifos degradation.

Term	Estimate	Standard Error	*t* Value	*P*r > | *t* | *
*X*_1_	−1.1843	1.1903	−0.9950	0.3379
*X*_2_	−7.5036	1.1903	−6.3041	0.0001
*X*_3_	7.8861	1.1903	6.6254	0.0001
*X*_1_ *X*_1_	−3.1131	1.1035	−2.8211	0.0144
*X*_1_ *X*_2_	−0.0950	1.5552	−0.0611	0.9522
*X*_1_ *X*_3_	0.3650	1.5552	0.2347	0.8181
*X*_2_ *X*_2_	−12.7085	1.1035	−11.5167	0.0001
*X*_2_ *X*_3_	1.1850	1.5552	0.7620	0.4597
*X*_3_ *X*_3_	−5.4890	1.1035	−4.9742	0.0002

* *P* Level less than 0.05 indicate the model terms are significant.

### Metabolic Products during Chlorpyrifos Degradation

The metabolic products of chlorpyrifos degradation by strain Hu-01 in mycelium-free filtrates were extracted and confirmed by GC-MS. The GC-MS results showed that two peaks at retention times (RT) of 15.135 and 9.140 min representing metabolites A and B, respectively.

**Figure 4:**
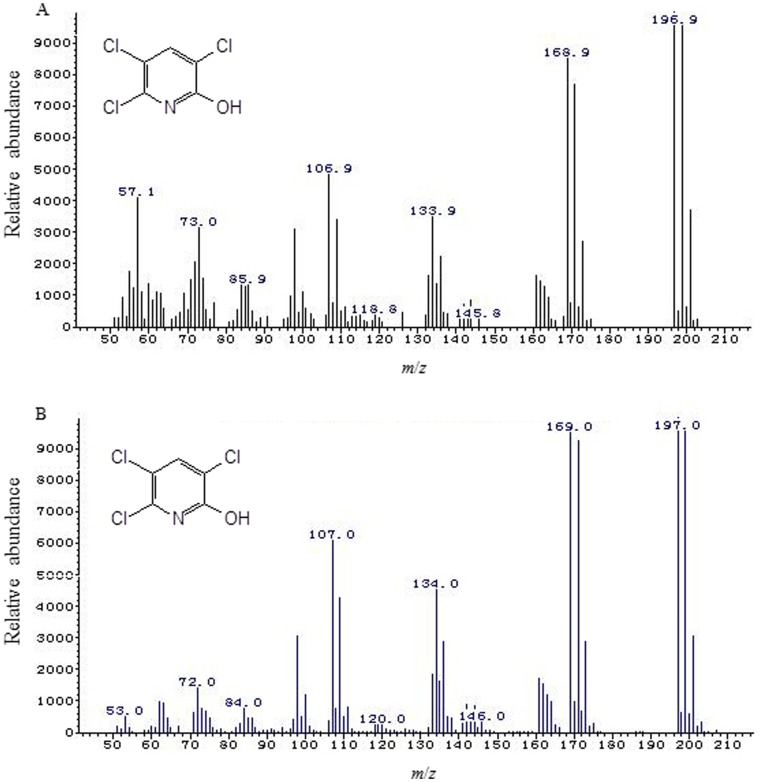
Mass spectra of 3,5,6-trichloro-2-pyridinol (TCP) produced from chlorpyrifos degradation by strain Hu-01. A: sample; B: authentic standard TCP from the National Institute of Standards and Technology (NIST, USA) library database.

Each of the two peaks was identified on the basis of its mass spectra and the NIST library identification program. The peak at retention time of 15.135 min corresponded to chlorpyrifos standard. This peak disappeared concomitantly with formation of another new peak with a retention time of around 9.140 min. Based on the characteristic fragment ion peaks and molecular ion (*m*/*z*), the new peak was identified as TCP (Fig. 4). However, this new peak was transient and it disappeared finally. Eventually, no persistent accumulative metabolite was detectable by GC-MS after 6 d of incubation.

**Figure 5:**
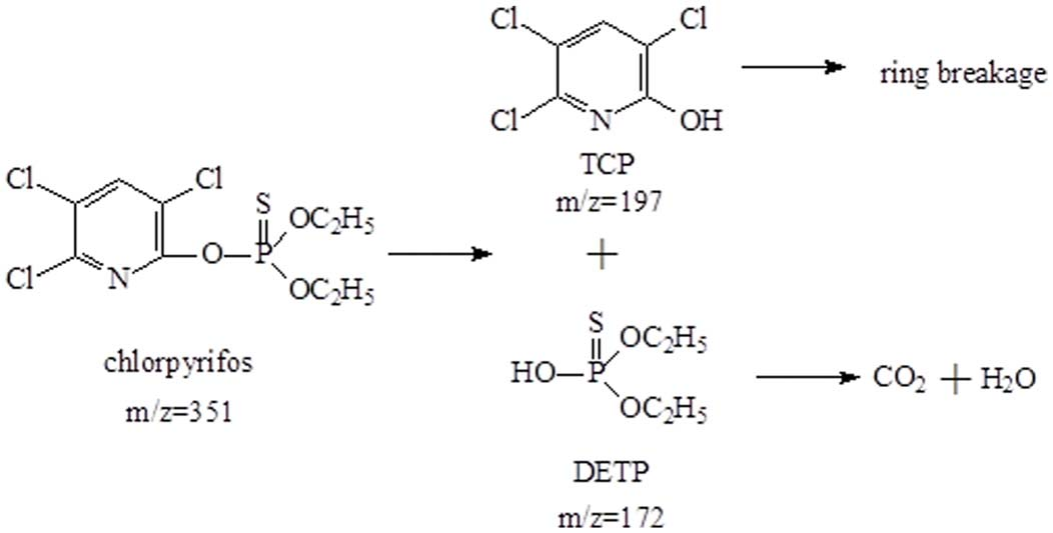
The proposed pathway for the chlorpyrifos degradation by strain Hu-01.

Based on the metabolic products formed, the degradation pathway for chlorpyrifos by strain Hu-01 was proposed (Fig. 5). That is to say, the parent chlorpyrifos (*m*/*z* = 351) was first metabolized by hydrolysis to produce TCP (*m*/*z* = 197) and diethylthiophosphoric acid (DETP) (*m*/*z* = 172). Subsequently, the hydrolysis product TCP was further transformed by ring breakage, resulting in its complete detoxification. These results thus indicated that the added chlorpyrifos (50 mg·L^−1^) was completely degraded by strain Hu-01 without any accumulative products after 6 d of incubation.

### Degradation Kinetics of Chlorpyrifos and TCP during Biodegradation Studies

**Figure 6:**
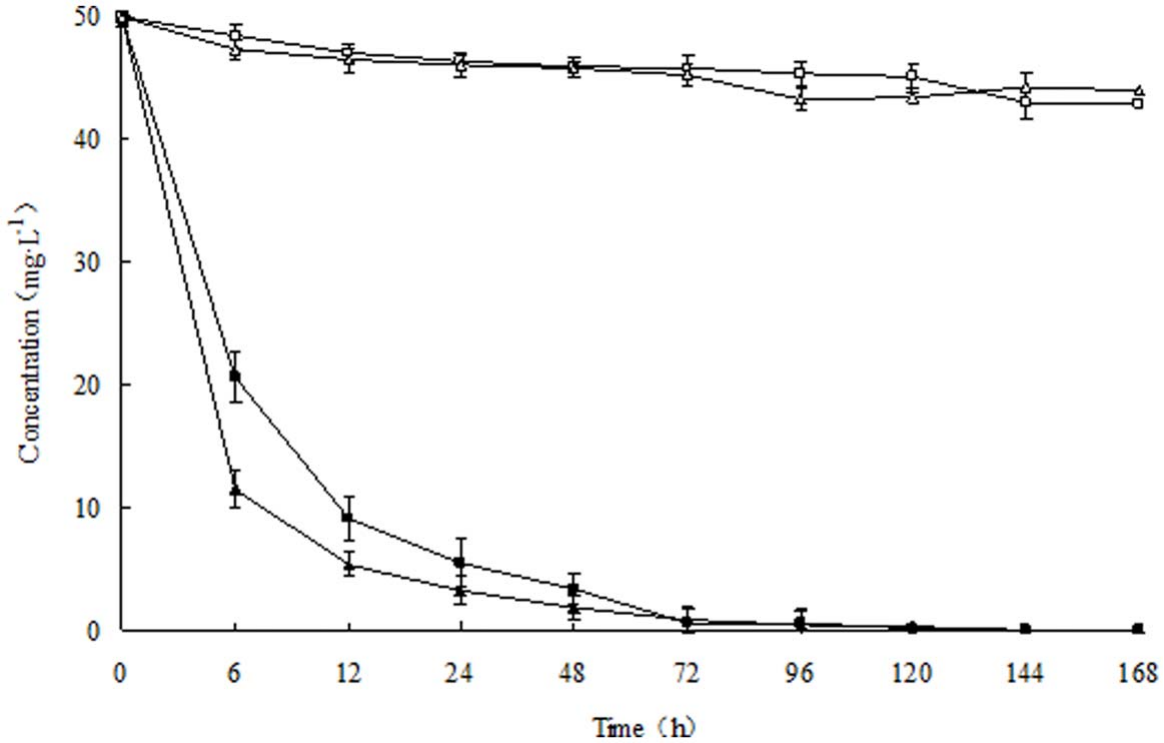
Degradation kinetics of chlorpyrifos and 3,5,6-trichloro-2-pyridinol (TCP) during biodegradation studies. □, non-inoculated control (chlorpyrifos); △, non-inoculated control (TCP); ▪, introduction with strain Hu-01 (chlorpyrifos); ▴, introduction with strain Hu-01(TCP). Error bars represent the standard deviation of three replicates.

The degradation kinetics of chlorpyrifos and its hydrolysis product TCP by strain Hu-01 were shown in Fig. 6. Introduction with strain Hu-01, rapid degradation was observed at the beginning of incubation, apparently there was no lag period and 89.0% of the 50 mg·L^−1^ chlorpyrifos initially added to the medium was degraded within 1 d. After 5 d of incubation complete degradation was achieved by the fungus; whereas the non-inoculated control this value decreased only by 14.4%. In the case of TCP, similar accelerated degradation was observed and 93.5% of the added TCP (50 mg·L^−1^) was eliminated within 1 d. After 6 d of incubation, there is no TCP detectable in the medium. In contrast, more than 88% of the added TCP remained in the medium that was not inoculated after completion of the experiment. During the chlorpyrifos degradation, trace amounts of TCP was also detected in the medium inoculated with strain Hu-01, but no TCP was found at the end of experiment. Therefore, incubation with strain Hu-01 resulted in greater chlorpyrifos and TCP degradation as compared to that in the non-inoculated controls.

To confirm the effects on biodegradation of chlorpyrifos and TCP by strain Hu-01, the first-order kinetic model (Eq.(5)) adapted from Cycoń et al. [3] was used to determine the rate of total chemical reduction
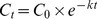
(5)where *C*_0_ is the initial concentration of chemical at time zero, *C*_t_ is the concentration of chemical at time *t*, *k* and *t* are the degradation rate constant (h^−1^) and degradation period in hours, respectively.

The algorithm as expressed in Eq.(6) was used to describe the theoretical half-life (*t*_1/2_) values of chlorpyrifos and TCP
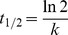
where ln2 is the logarithmic function, *k* is the rate constant (h^−1^).

The kinetic parameters for all runs calculated from the above equations (Eq.(5) and Eq.(6)) are presented in Table 5. In these studies, the degradation rate and the chemical concentration were in direct proportion, and the degradation process corresponded to the first-order kinetics with a *R* ^2^ ranging from 0.9165 to 0.9904, revealing that the experimental data fit well with the model. The *k* for chlorpyrifos and TCP with strain Hu-01 were 0.1398 and 0.2201 h^−1^, respectively; while the control the *k* were 0.0010 and 0.0007 h^−1^, respectively. The *t*_1/2_ for chlorpyrifos and TCP with strain Hu-01 were 5.0 and 3.1 h, respectively. In contrast, the *t*_1/2_ for chlorpyrifos and TCP in the non-inoculated controls were 693.0 and 990.0 h, respectively. Compared with the controls, the *t*_1/2_ for chlorpyrifos and TCP greatly reduced by 688.0 and 986.9 h, respectively. These results thus confirmed that inoculation with strain Hu-01 significantly enhanced degradation of chlorpyrifos and TCP. At the end of the reaction, both pollutants were successfully eliminated by the fungal strain *C. cladosporioides* Hu-01.

**Table 5:** Kinetic parameters of degradation of chlorpyrifos and 3,5,6-trichloro-2-pyridinol (TCP) by strain Hu-01.

Treatments	Regression equation	*k* (h^−1^)	*t*_1/2_ (h)	*R* ^2^
Chlorpyrifos	*C_t_* = 48.4009e^−0.0010*t*^	0.0010±0.0002c	693.0±51.6b	0.9244
TCP	*C_t_* = 48.5899e^−0.0007*t*^	0.0007±0.0001c	990.0±79.9a	0.9165
Chlorpyrifos + Hu-01	*C_t_* = 49.6522e^−0.1398*t*^	0.1398±0.0019b	5.0±0.6c	0.9855
TCP + Hu-01	*C_t_* = 47.8202e^−0.2201*t*^	0.2201±0.0035a	3.1±0.4c	0.9904

+ refers to introduced with; *C_t_*: residual concentration of chemicals (mg·kg^−1^); *t*: degradation period (h). *R* ^2^: correlation coefficient. The data presented are means of three replicates with standard deviation. Data followed by the same letters in the same column are not significantly different at *P* = 0.05 level according to the Tukey’s test.

## Discussion

Given the widespread chlorpyrifos use around the world and prevalent human exposure, effective techniques for disposal of this pollutant are critically needed [6], [9], [53], [54]. Bioremediation provides a cheap and environmentally friendly way to remove toxic pollutants from the environment [2]. In the current study, a novel fungal strain *C. cladosporioides* strain Hu-01 responsible for chlorpyrifos degradation was isolated from organophosphate-contaminated soils using an enrichment procedure. This particular isolate completely degraded not only chlorpyrifos but also its hydrolysis product TCP. This feature is rarely reported in other chlorpyrifos-degrading microorganisms. In most cases reported to date, the individual isolate tended to transform chlorpyrifos by hydrolysis of ester linkage to yield TCP, which in turn accumulated in the batch cultures or soils and enhanced degradation could not occur owing to its antimicrobial properties [4], [28], [29], [30], [32]. TCP is associated with estrogenic activity and has recently been listed as potential endocrine disrupting chemical by the Environmental Protection Agency (EPA) of the USA [55]. Compared with the importance of TCP degradation issue, studies concerning its fate and degradation in the environment are very limited. *Ralstonia* sp. strain T6 was reported to metabolize 100 mg·L^−1 ^TCP within 12 h, but it could not transform chlorpyrifos [30]. As far as we know, this is the first report about a fungal strain involving in chlorpyrifos and TCP degradation.

There has been some success in the use of degrading bacteria for bioremediation of chlorpyrifos. For example, Dubey and Fulekar [56] demonstrated that *Stenotrophomonas maltophilia* strain MHF ENV20 isolated from rhizosphere played an important role in reducing chlorpyrifos level in soil. Plasmid-mediated bioaugmentation for the enhanced biodegradation of chlorpyrifos in soil was recently achieved by the indigenous bacteria, including members of the *Pseudomonas* and *Staphylococcus* genera [34]. Unfortunately, relative contributions of fungi to degradation processes have seldom been quantified. Fungi possess the biochemical and ecological capacity to detoxify environmental xenobiotics, either by chemical modification or by influencing chemical bioavailability [36]. However, despite dominating the living biomass in soil and being abundant in aqueous systems, fungi have not received the attention they deserve. Though several fungal strains have been reported to degrade chlorpyrifos, yet attempts to isolate fungi in pure culture able to mineralize chlorpyrifos have often failed [36]–[38], [57]. For example, mineralization of chlorpyrifos (50 mg·L^−1^) was achieved by co-culture of *Serratia* sp. TCR and *Trichosporon* sp. TCF, but the pure fungal strain TCF alone could not transform chlorpyrifos [57].

In order to obtain efficient degradative fungi, in the last few years our laboratory has screened a wide range of contaminated soils from organophosphate-manufactuer system in China for chlorpyrifos degradation activity. A fungal strain, designated Hu-01, capable of completely degrading chlorpyrifos and its hydrolysis product TCP was successfully isolated from the soil. 5.8S rDNA gene sequencing and the morphological characteristics strongly revealed that strain Hu-01 belongs to the genus *Cladosporium*. To the best of our knowledge, there is no information regarding the ability to metabolize organophosphorous pesticides by fungal strain belonging to the genus *Cladosporium*. Nevertheless, several studies have suggested that *Cladosporium* species have some potential bioactivity, such as antimicrobial activity and the ability to degrade aromatic compounds [47], [58], [59].

Notably, *C. cladosporioides* strain Hu-01 was found to efficiently degrade chlorpyrifos over a broad range of temperature and pH particularly at low pH, as summarized in Table 2. This feature gives pesticide degraders advantages in the variable environments, because they survival and utilize xenobiotic compounds even exposed to adverse conditions. In contrast, previous studies showed that rapid degradation of chlorpyrifos by *Enterobactor* sp. was observed at high pH while this activity was very slow at acidic pH [60]. Anwar et al. [4] reported that chlorpyrifos was more efficiently degraded at basic as well as neutral pH whereby more than 80% of the added pesticide was degraded by *Bacillus pumilus* strain C2A1 and no lag phase was observed at relatively higher pH. However, at acidic pH only 50% degradation was observed with longer lag phase. Our results contrasted with previous findings and the chlorpyrifos degradation by strain Hu-01 was higher at the acidic conditions. It is possible that some key enzyme(s) responsible for chlorpyrifos degradation have their optimum enzymatic activity at relatively low pH value. Another important feature which is worth mentioning is that this strain engaged in efficient degradation of chlorpyrifos and its hydrolysis product TCP up to the concentrations, as high as 500 mg·L^−1^. In contrast to other reports on toxic effects of chlorpyrifos on diverse microorganisms [28], [29], [61], our results indicated that chlorpyrifos metabolism activity of strain Hu-01 was not subject to complete catabolite repression by high chlorpyrifos concentrations. High chlorpyrifos tolerance and degradation capability of *C. cladosporioides* strain Hu-01, makes this strain suitable for decontamination and remediation of contaminated sites.

In this study, the optimum culture conditions for chlorpyrifos degradation by *C. cladosporioides* strain Hu-01 were determined by using response surface methodology (RSM). RSM is an empirical modeling system that has been successfully applied to improve and optimize complex processes, including fermentation processes and medium components for a variety of microorganisms [50], [51]. Previous studies have shown that the application of statistical experimental design techniques in biodegradation processes can result in improved yields of degradation and allow the rapid and economical determination of the optimum culture conditions with fewer experiments and minimal resources [47], [49], [62]–[65]. In the present study, RSM is first employed to optimize culture conditions favouring chlorpyrifos degradation. The optimization parameters investigated include pH, temperature and culture time. The results of the experiments were statistically analyzed and the significance and effect of each factor on responses was evaluated. The optimum degradation conditions were determined to be pH 6.5, 26.8°C, and culture time 4.7 d. Under these conditions, strain Hu-01 reached a maximum chlorpyrifos degradation of approximately 85% within 12 h. Moreover, a mathematical model (Eq.(4)) was developed herein, and this model could be effectively used to predict and optimize the chlorpyrifos degradation conditions by strain Hu-01 within the limits of chosen factors.

It was generally considered that ester hydrolysis was the primary degradation pathway of chlorpyrifos [66], [67]. In most cases, the degrading microorganisms tend to metabolize chlorpyrifos by hydrolysis to form diethylthiophosphoric acid (DETP) and TCP [4], [29], [31], [32]. However, due to its resistant to enhanced degradation, studies on further metabolism and identification of intermediate products of chlorpyrifos have not been extensive [29]. In our studies, degradation of chlorpyrifos was accompanied by a transient accumulation of TCP. Moreover, the only intermediate product disappeared quickly. Obviously, TCP was further transformed without any other persistent metabolites by strain Hu-01. Finally, no persistent accumulative metabolite was detected by GC-MS after 6 d of incubation. Some other metabolites might have formed and been immediately degraded by the fungus. In our previous work, we have purified the chlorpyrifos hydrolase from the fungus [52]. Based on these results, the metabolic pathway of chlorpyrifos was first proposed (Fig. 5).

Previous studies suggested that the conditions for environmental microorganism enrichment and screening are of vital importance in the selection of pure isolates with high survivability in the environment and maximum degrading activity towards xenobiotics [29]. However, it has been problematic to isolate a pure isolate capable of utilizing chlorpyrifos and TCP for growth substrates [4]. Several reported microorganisms degraded chlorpyrifos and TCP co-metabolically, which needed extra carbon sources for enhanced degradation [68], [69]. In our studies, the fungal strain isolated from the contaminated soils appeared to be highly efficient in degrading chlorpyrifos and TCP in MSM without any other carbon sources and utilizing these pollutants as the sole carbon sources. It could utilize 89.0% and 93.5% of the added chlorpyrifos and TCP (both 50 mg·L^−1^) within 1 d, respectively (Fig. 6). The *t*_1/2_ for chlorpyrifos and TCP greatly reduced by 688.0 and 986.9 h, respectively as compared to the controls (Table 5). These results demonstrated the isolate may be suitable for the efficient and rapid bioremediation of contaminated environments.

In conclusion, the *C. cladosporioides* strain Hu-01 isolated in the present study completely metabolized not only chlorpyrifos but also its hydrolysis product TCP. This is the first report involving in the biodegradation of both chlorpyrifos and TCP by a fungal strain from the genus *Cladosporium*. Moreover, metabolization of chlorpyrifos and TCP by the same strain is of vital importance because TCP possesses antimicrobial activities, and it tends to accumulate in the batch cultures or soils and hence prevents the proliferation of microorganisms responsible for the parent compound degradation through catabolite repression. In addition, strain Hu-01 was found highly efficient in degrading chlorpyrifos over a wide range of temperature and pH particularly at low pH. This feature gives pesticide degraders advantages in the variable environments. Another important feature which is worth mentioning is that this strain harbors the metabolic pathway for the detoxification of chlorpyrifos, and it completely degraded chlorpyrifos without any persistent accumulative metabolites. Furthermore, the fungus utilized chlorpyrifos and TCP as the sole carbon sources for growth, thus suggesting adaptation to oligotrophic environments. The ability to survive at high concentration of chlorpyrifos and enhanced degradation make this isolate an ideal candidate for its application in chlorpyrifos bioremediation.

## Supporting Information

Figure S1 Morphological characteristics of strain Hu-01 under scanning electron microscope. (3,200×).
(TIF)

Table S1 The degradation of 50 mg·L^−1^ chlorpyrifos in MSM by all the isolated strains by day 5.
(DOC)
