# Supplementary figures and images for: Biodegradation of Chlorpyrifos and Its Hydrolysis Product 3,5,6-Trichloro-2-Pyridinol by a New Fungal Strain Cladosporium cladosporioides Hu-01
Source: PLoS One. 2012 Oct 8;7(10):e47205. doi: 10.1371/journal.pone.0047205 (PMC3466218; doi:10.1371/journal.pone.0047205)

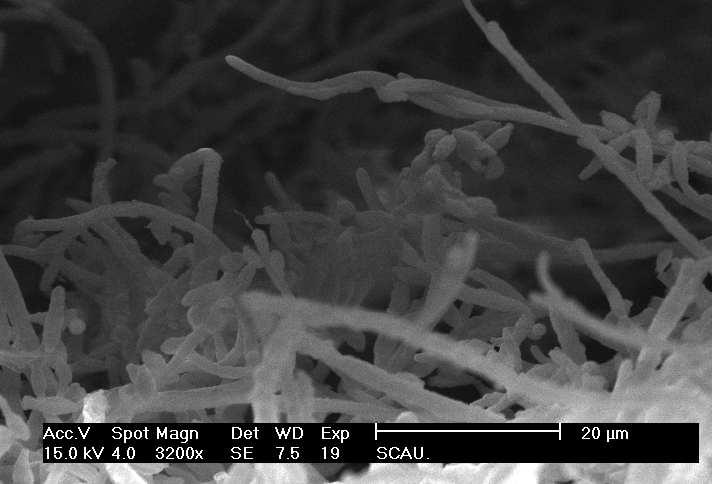

Supplement: Figure S1 — Morphological characteristics of strain Hu-01 under scanning electron microscope. (3,200×). (TIF) [file pone.0047205.s001.tif]
