# Supplementary material for: Biodegradation of Chlorpyrifos and Its Hydrolysis Product 3,5,6-Trichloro-2-Pyridinol by a New Fungal Strain Cladosporium cladosporioides Hu-01
Source: PLoS One. 2012 Oct 8;7(10):e47205. doi: 10.1371/journal.pone.0047205 (PMC3466218; doi:10.1371/journal.pone.0047205)
Supplement: Table S1 — The degradation of 50 mg·L−1 chlorpyrifos in MSM by all the isolated strains by day 5. (DOC) [file pone.0047205.s002.doc]

**Table S1**

The degradation of 50 mg·L^-1^ chlorpyrifos in MSM by all the isolated strains by day 5

| Strains | Degradation rate (%) |
| --- | --- |
| Hu-01  Hu-02  Hu-03  Hu-04  Hu-05  Hu-06  Hu-07  Hu-08 | 100±0a  73.2±2.2b  70.5±2.0b  64.1±1.1b  50.6±2.8c  38.6±1.7d  24.9±0.6e  11.3±1.0f |

The data presented are means of three replicates with standard deviation. Data followed by the same letters in the same column are not significantly different at *P* = 0.05 level according to the Tukey’s test.
